# Early response monitoring during [^177^Lu]Lu-PSMA I&T therapy with quantitated SPECT/CT predicts overall survival of mCRPC patients: subgroup analysis of a Swiss-wide prospective registry study

**DOI:** 10.1007/s00259-023-06536-2

**Published:** 2023-12-01

**Authors:** Moritz C. Neubauer, Guillaume P. Nicolas, Andreas Bauman, Melpomeni Fani, Egbert Nitzsche, Ali Afshar-Oromieh, Flavio Forrer, Cyril Rentsch, Frank Stenner, Arnoud Templeton, Niklaus Schäfer, Damian Wild, Alin Chirindel, all investigators on behalf of the SSNM Therapy Working Group

**Affiliations:** 1 Clinic for Radiology and Nuclear Medicine, University Hospital Basel, Petersgraben 4, 4051 Basel, Switzerland; 2 Nuclear Medicine and PET-Center, Cantonal Hospital Aarau, Aarau, Switzerland; 3 Nuclear Medicine, University Hospital Bern, Bern, Switzerland; 4 Nuclear Medicine, Cantonal Hospital St. Gallen, St. Gallen, Switzerland; 5 Urology, University Hospital Basel, Basel, Switzerland; 6 Oncology, University Hospital Basel, Basel, Switzerland; 7 Oncology, St. Claraspital, Basel, Switzerland; 8 Nuclear Medicine, University Hospital Lausanne, Lausanne, Switzerland

**Keywords:** Metastatic prostate cancer, [^177^Lu]Lu-PSMA I&T, Quantitative SPECT/CT, Response monitoring

## Abstract

**Purpose:**

To assess early tumor response with quantitated SPECT/CT and to correlate it with clinical outcome in metastatic castration–resistant prostate cancer (mCRPC) patients treated with ^177^Lutetium-PSMA I&T therapy.

**Methods:**

Single-center, observational study, part of the prospective Swiss national cancer registry study investigating the safety and efficacy of [^177^Lu]Lu-PSMA I&T (EKNZ: 2021–01271) in mCRPC patients treated with at least two cycles of [^177^Lu]Lu-PSMA I&T 6-weekly. After the first and second cycle quantitated SPECT/CT (Symbia Intevo, Siemens) was acquired 48 h after injection (three fields of view from head to thigh, 5 s/frame) and reconstructed using xQuant® (48i, 1 s, 10-mm Gauss). Image analysis: The PSMA-positive total tumor volumes (TTV) were semi-automatically delineated using a SUV threshold of 3 with MIMencore® (version 7.1.3, Medical Image Merge Software Inc.). Changes in TTV, highest tumor SUVmax, and total tumor SUVmean between cycles 1 and 2 were calculated and grouped into a) stable or decrease and b) increase. Serum PSA levels were assessed at each therapy cycle and at follow-up until progression or death. Changes in TTV, PSA, SUVmax, and SUVmean were correlated with PSA-progression-free survival (PSA-PFS) and the overall survival (OS) using the Kaplan–Meier methodology (log-rank test).

**Results:**

Between 07/2020 and 04/2022, 111 patients were screened and 73 finally included in the data analysis. The median follow-up was 8.9 months (range 1.4–26.6 months). Stable or decreased TTV at cycle 2 was associated with longer OS (hazard ratio (HR) 0.28, 95% confidence interval (CI) 0.09–0.86, *p* < 0.01). Similar, stable, or decreased PSA was associated with longer OS (HR 0.21; CI 0.07–0.62, *p* < 0.01) and PSA-PFS (HR 0.34; 95% CI 0.16–0.72, *p* < 0.01). Combining TTV and PSA will result in an augmented prognostic value for OS (HR 0.09; CI 0.01–0.63; *p* < 0.01) and for PSA-PFS (HR 0.11; CI 0.02–0.68; *p* < 0.01). A reduction of SUVmax or SUVmean was not prognostically relevant, neither for OS (*p* 0.88 and 0.7) nor for PSA-PFS (*p* 0.73 and 0.62, respectively).

**Conclusion:**

Six weeks after initiating [^177^Lu]Lu-PSMA I&T, TTV and serum PSA appear to be good prognosticators for OS. Combined together, TTV + PSA change demonstrates augmented prognostic value and can better predict PSA-PFS. Larger studies using TTV change prospectively as an early-response biomarker are warranted for implementing management change towards a more personalized clinical practice.

**Supplementary Information:**

The online version contains supplementary material available at 10.1007/s00259-023-06536-2.

## Introduction

Targeted radioligand therapy (RLT) with Lutetium-177-prostate-specific membrane antigen ligand (^177^Lu-PSMA) has emerged as a new and effective treatment option in patients with metastatic castration–resistant prostate cancer (mCRPC).

To date, the two most used PSMA-targeting small-molecule inhibitors for PSMA-targeted RLT are [^177^Lu]Lu-PSMA-617 (Pluvicto) and [^177^Lu]Lu-PSMA I&T which, although different in their chemical structure, exhibit comparable organ biodistribution and rather similar tumor dose [1]. Based on the randomized multicenter phase III trial VISION [2], Pluvicto ([^177^Lu]Lu-PSMA-617) was approved by the FDA on March 23, 2022, for mCRPC patients previously treated with androgen receptor pathway inhibitors and taxane-based chemotherapy. Further phase III trials are currently being conducted in earlier therapy timelines for prostate cancer for both [^177^Lu]Lu-PSMA-617, PSMAfore trial (NCT04689828) and the PSMAddition trial (NCT04720157), and [^177^Lu]Lu-PSMA I&T, SPLASH (NCT04647526) and ECLIPSE (NCT05204927).

However, treatment failure and particularly short response duration are ongoing significant challenges in prostate RLT, which is still dominated by a continual lack of consensus among specialists on the most appropriate therapy protocol, including the applied activity, the number and pace of ^177^Lu-PSMA cycles, and the response monitoring during therapy [1–3]. The latter is quite relevant as by recognizing early treatment failure, improved and personalized treatment options could be instituted (e.g., by reducing unnecessary therapy cycles and their associated risk of adverse events as well as reducing costs and improving patient care by initiating an earlier change in treatment if available).

Prior reports have reported a good correlation between changes in the total PSMA-positive tumor volume (TTV) under therapy and clinical outcome; however, they differ in their used imaging modality (PET/CT or SPECT/CT) as well as compared time points (6 weeks or 12 weeks after start of therapy) and used compound ([^177^Lu]Lu-PSMA-617 or [^177^Lu]Lu-PSMA I&T) [4–6].

Given that data regarding SPECT/CT-based treatment response monitoring are scarce and heterogeneous, the primary goal of this study was to assess the value of quantitative whole-body tumor volumetry performed on SPECT/CT imaging at 48 h after administration of [^177^Lu]Lu-PSMA I&T therapy in mCRPC patients.

Second, this will generate a quantitative dataset for treatment response monitoring in [^177^Lu]Lu-PSMA I&T RLT, complementary to the recently presented data regarding treatment response evaluation derived from imaging at 24 h after therapy initiation of [^177^Lu]Lu-PSMA I&T [4].

## Material and methods

### Patient selection

This was a single-center, retrospective study done at the University Hospital Basel as part of a national Swiss-wide cancer registry study analyzing data from 07/2020 to 04/2022. Data collection was part of a national registry study approved by the Swiss Federal Office of Public Health (EKNZ: 2021–01271).

### Treatment

[^177^Lu]Lu-PSMA I&T (mean dose 6.42 GBq, SD 1.44 GBq) was administered according to the established protocol every 6 weeks therapy analogue to the VISION trial protocol [3].

### Imaging

Quantitated SPECT/CT (Symbia Intevo, Siemens, Munich, Germany) was acquired about 48 h (mean 42 h, SD 1.7 h, range 40–52 h) after injection (5 s/frame) and reconstructed using xQuant® (48i, 1 s, 10-mm Gauss).

### Imaging analysis

**Fig. 1:**
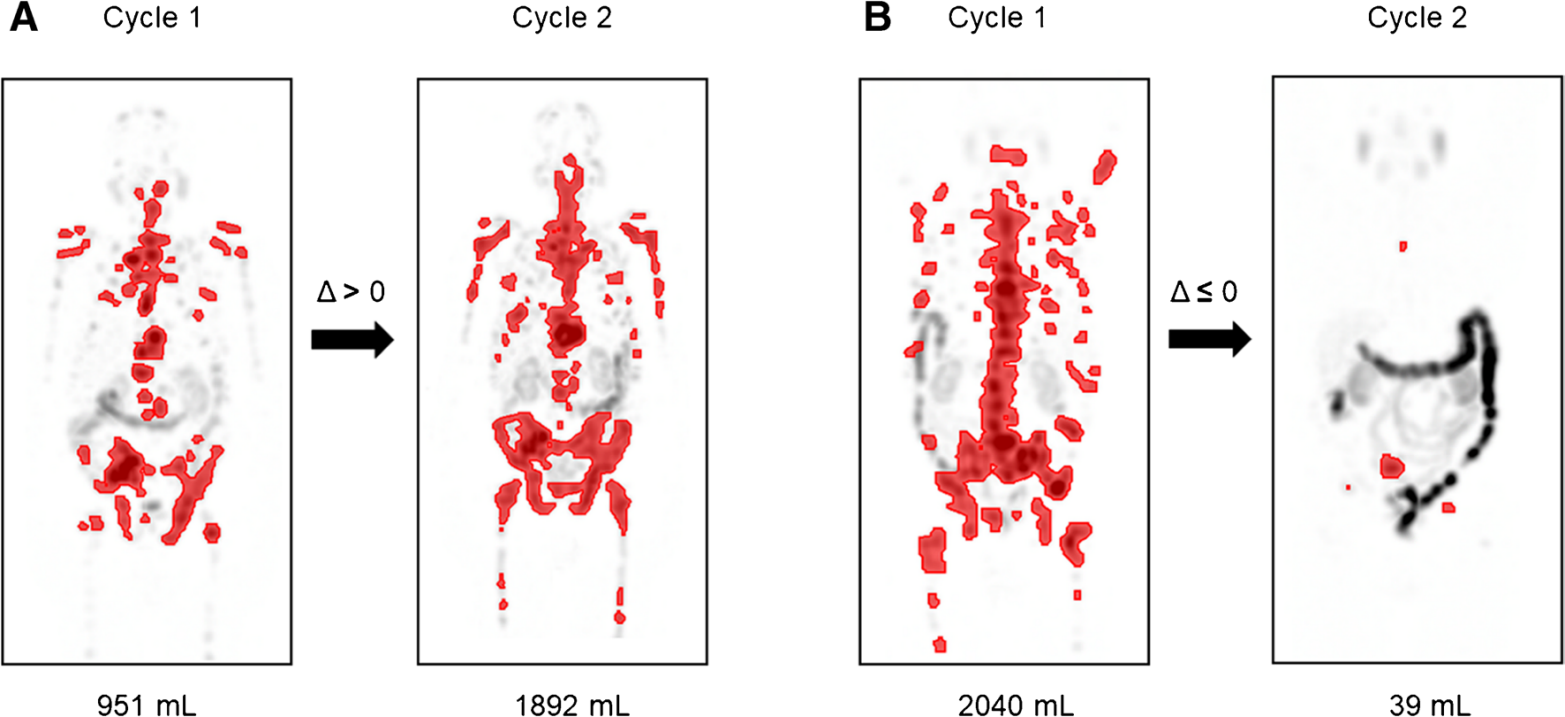
Exemplary SPECT-MIP images of two patients 48 h after the first and second injections. Semiautomated tumor volumetry (given in mL) done with MIMencore (SUV threshold = 3). **A** Non-responder defined by any increase in TTV (Δ > 0 mL) and **B** responder defined as stable or decrease in TTV (Δ ≤ 0 mL)

Tumor delineation was done semi-automatically by MIMencore® (version 7.1.3, Medical Image Merge Software Inc., Cleveland, USA) using the “Lesion ID” workflow protocol. Physiological activity was manually removed by a blinded reader not aware of any clinical information, especially the patient’s outcome. The TTV, highest tumor SUVmax, and SUVmean were delineated using a SUV threshold of 3. According to their change in TTV or highest tumor SUVmax, patients were grouped into (a) responder defined as change ≤ 0 or (b) non-responder, defined as change > 0 (Fig. 1).

### Clinical endpoints

OS and PSA-PFS were measured from the start of the first cycle. PSA-PSF was determined as defined by the Prostate Cancer Clinical Trials Working Group 3 (PCGW3 [7]) with an increase in PSA greater than 25% and > 2 ng/mL above nadir, confirmed by PSA progression at two sequential time points at least 3 weeks apart.

For a subgroup analysis with only two time points, a PSA rise between cycles 1 and 2 was solely defined as an increase of PSA ≥ 2 ng/mL, similar to previous studies [4].

PSA-PFS and OS between the two groups were compared using the Kaplan–Meier methodology (log-rank test). Univariate and multivariate analyses with all possible predictors and confounding factors were performed using a Cox proportional hazards regression model. Statistical analysis and graph artwork were done using GraphPad Prism® (version 9.4.1, GraphPad Software, San Diego, USA).

## Results

**Fig. 2:**
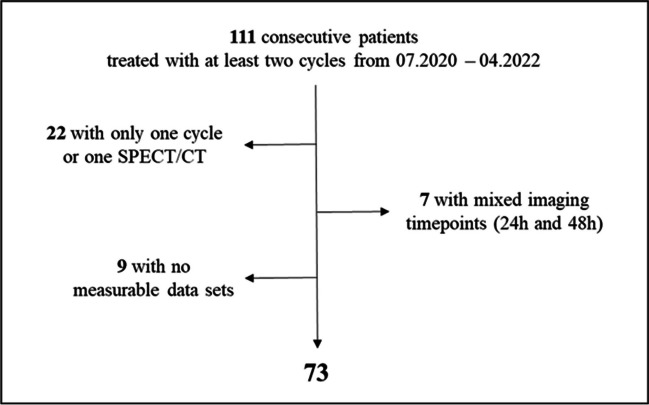
Patient flowchart

The total patient cohort consisted of 111 consecutive male mCRPC patients who underwent ^177^Lu-PSMA radioligand therapy. Of those patients, we only included patients with at least two cycles with ^177^Lu-SPECT/CT done 48 h after intravenous administration of [^177^Lu]Lu-PSMA I&T (*n* = 82). In nine patients, the acquired data set could not be processed due to technical reasons. The final patient cohort consisted of 73 patients (Fig. 2) with patient characteristics as depicted in Table 1.

**Table 1:** Patient’s characteristics at the start of therapy. Systemic radionuclide therapies include xofigo and PSMA-targeted RLT

Patients characteristics	*N* = 73
Age	Mean 75 y, range 57–91 y
Years since diagnosis	Mean 8.1 y, range 0.6–24.7 y
Gleason-score (when available)	Mean 8, range 6–10
Pretreatment regimes:	
Local surgical tumor resection	32 (44%)
Antiandrogen therapy	71 (97%)
Taxane-based chemotherapy	47 (64%)
Salvage-radiation	13 (18%)
Anti-androgen receptor therapies	67 (92%)
Systemic radionuclide therapy	14 (19%)
Palliative external beam radiotherapy	45 (62%)
Metastasis:	
Bone	69 (95%)
Nodal	57 (78%)
Visceral	18 (25%)

### Total tumor volume (TTV)

Fifty-five patients (75%) showed a stable or reduced TTV at cycle 2 and were defined as responders. Of those, 37 had a documented PSA progression and 12 died. Eighteen patients (25%) had an increase in TTV, 13 of them with a PSA progression, and 8 patients died.

Compared to the non-responders (defined as any increase in TTV), the responder group showed a significant increase in OS (*p* value 0.0017; hazard ratio (HR) 0.28, 95% confidence Interval (CI) 0.09–0.86) and no significant increase in PSA-PFS (*p* value 0.0700; HR 0.51, 95% CI 0.25–1.06).

**Fig. 3:**
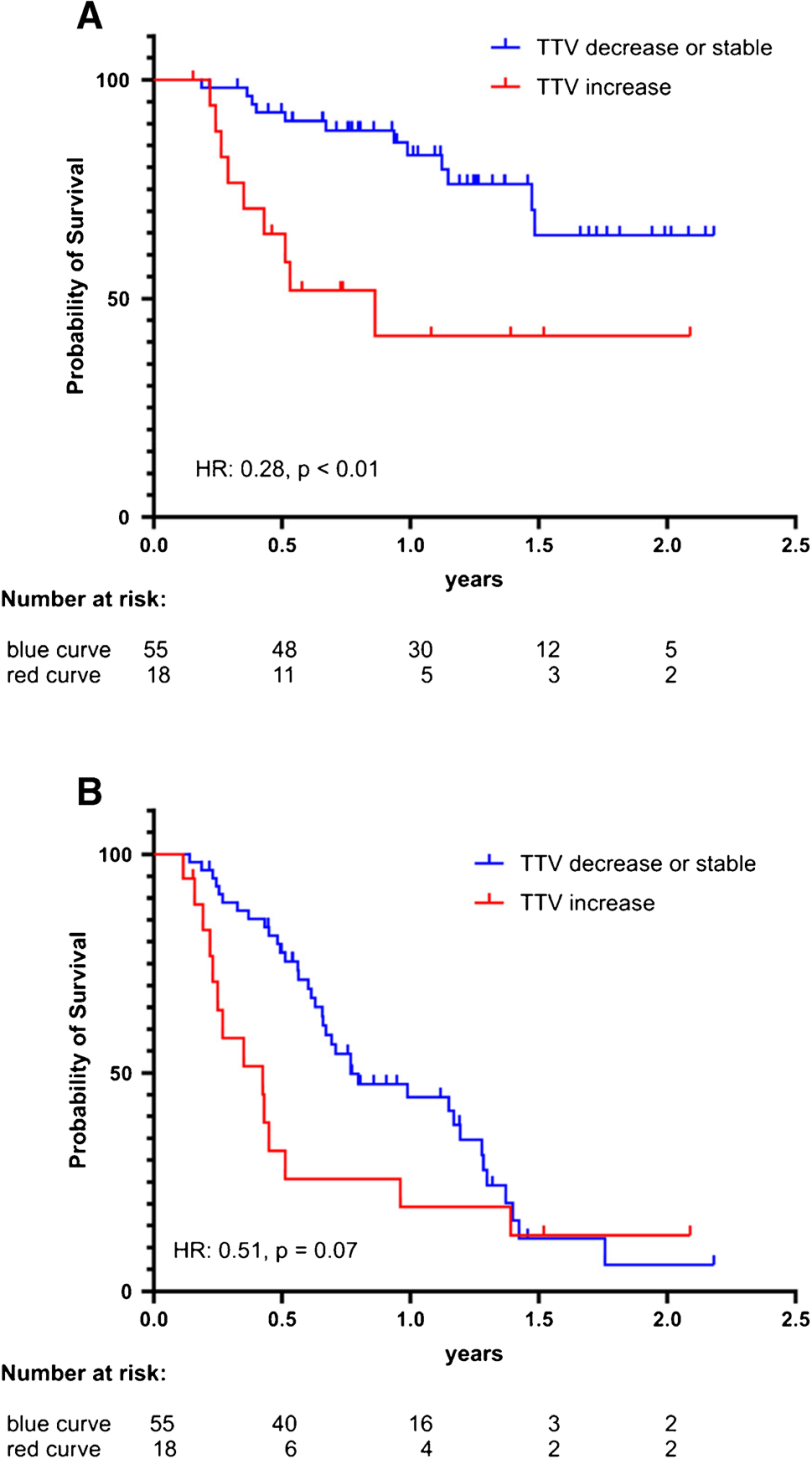
Kaplan–Meier curves with log-rank (Mantel-Cox) test *p* values: **A** OS and **B** PSA-PFS compared between patients with increased (> 0 mL) to stable or decreased (≤ 0 mL) TTV. Compared to the non-responder group (TTV increase, red), the responder group (TTV stable or decrease, blue) showed a significant increase in OS (*p* value 0.0017, HR 0.28) and no significant increase in PSA-PFS (*p* value 0.0700; HR 0.51)

For the non-responder group, median PSA-PFS was 5.1 months, and median OS was 10.3 months, whereas for the responder group, median PFS was 9.2 months, and median OS could not be reached (Fig. 3).

In the multivariate Cox regression analysis, TTV change remained statistically significant (see Table 1 in the supplemental material). Moreover, TTV change continued to be relevant for overall survival when tested together with metastatic pattern as possible cofounder.

### Highest tumor SUVmax and total tumor SUVmean

Fifty-nine patients (81%) had a stable or reduced highest tumor SUVmax, 40 of these had a documented PSA progression, and 16 died. Fourteen patients (19%) had an increase in the highest tumor SUVmax, 11 of them had a documented PSA progression, and 4 of them died.

An absolute reduction of the highest SUVmax was not correlated with longer OS (*p* value 0.8788; HR 1.088, 95% CI 0.38–3.15) or PSA-PFS (*p* value 0.7308; HR 0.89, 95% CI 0.45–1.78), respectively. With only slight differences, these findings also applied for SUVmean (OS *p* value 0.6965, HR 0.81, 95% CI 0.25–2.59 and PSA-PFS *p* value 0.6214, HR 0.84, 95% CI 0.39–1.80).

We further investigated the effect of the total lesion activity, measured as a product of the total tumor SUVmean and the total tumor volume (SUVmean*TTV). Though the SUVmean*TTV product at baseline was neither associated with OS nor with PSA-PFS, an increase from cycle 1 to cycle 2 was significantly associated with shorter OS in a univariate analysis (HR 2.93; 95% CI 1.40–5.81, *p* = 0.0027).

### PSA

Nineteen patients (26%) had a rise in PSA levels (> 2 ng/mL) between baseline and 6 weeks after. Out of these, 11 died and 18 had a confirmed PSA progression.

Thirty-two patients (44%) were considered stable in their PSA levels; 10 of them had only slightly rising PSA levels (Δ < 2 ng/mL), and 22 patients had sinking PSA levels, yet below 50% of their baseline (PSA50 not reached). Twenty-two patients (30%) had a decrease in PSA ≥ 50% of baseline (PSA50). Of these 54 patients with stable and decreased PSA levels, 10 died and 33 had a documented PSA progression.

**Fig. 4:**
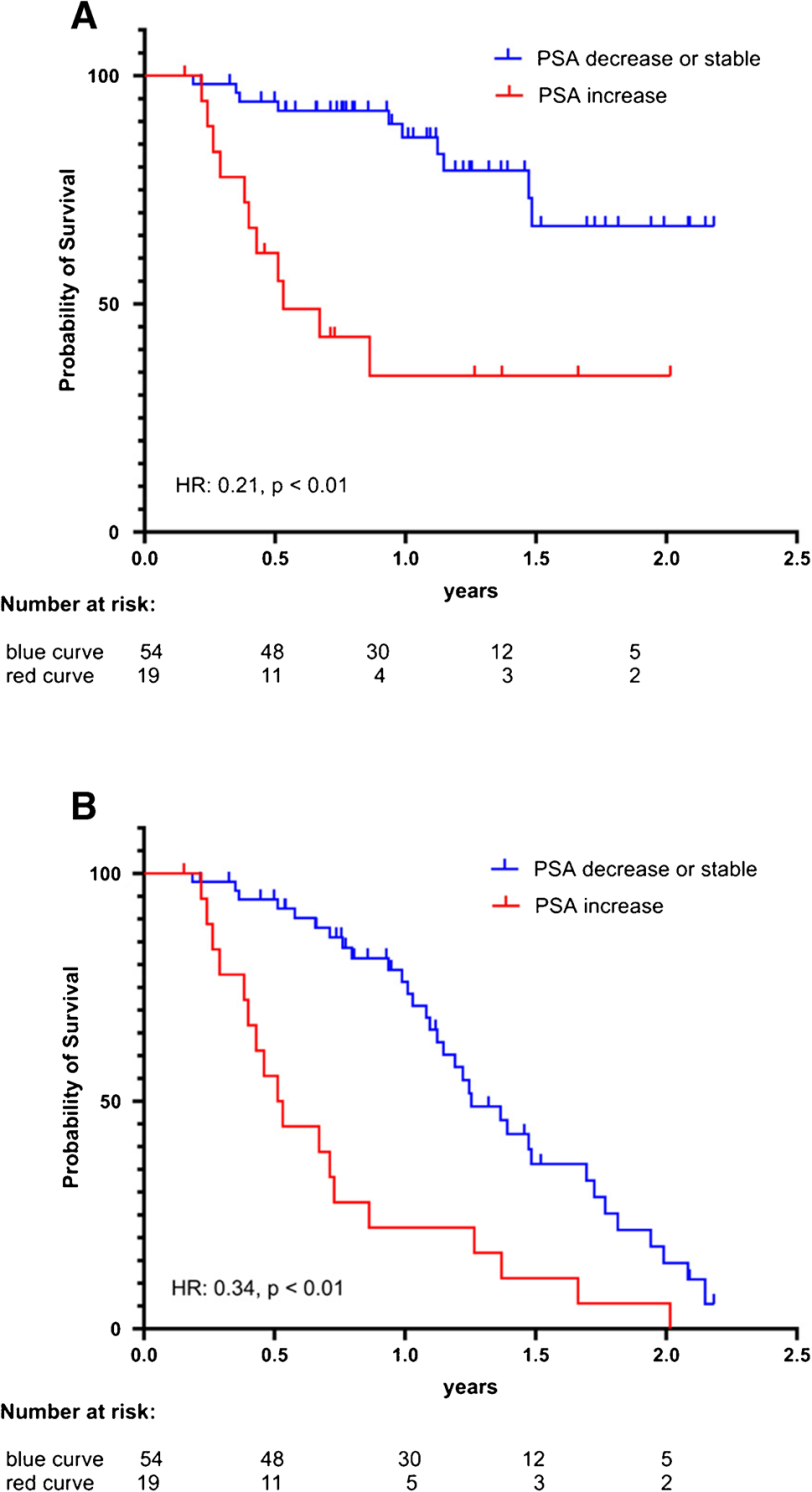
Kaplan–Meier curves with log-rank (Mantel-Cox) test *p* values: **A** OS and **B** PSA-PFS compared between patients with a PSA rise between the first and second cycle (Δ ≥ 2 ng/mL) to patients with decreased (≥ 50% reduction) or stable PSA levels (between < 50% reduction and a rise < 2 ng/mL). A stable or reduced PSA level after 6 weeks of therapy (blue) was associated with a significant longer OS (*p* < 0.0001; HR 0.21) and PSA-PFS (*p* < 0.0001; HR 0.34) compared to an increased PSA level (red)

A stable or reduced PSA level after 6 weeks of therapy was associated with a significant increase in OS (*p* < 0.0001, HR 0.21; 95% CI 0.07–0.62) and PSA-PFS (*p* < 0.0001; HR 0.34; 95% CI 0.16–0.72). For the group with rising PSA values, the median PSA-PFS and median OS were 6.3 and 6.4 months, respectively, whereas for the stable or decreased group, the median PSA-PFS was 15 months, and median OS could not be reached (Fig. 4). Similar to TTV, in our multivariate Cox regression analysis, PSA change remained statistically significant (see Table 1 in supplemental material).

### TTV grouped with PSA

**Fig. 5:**
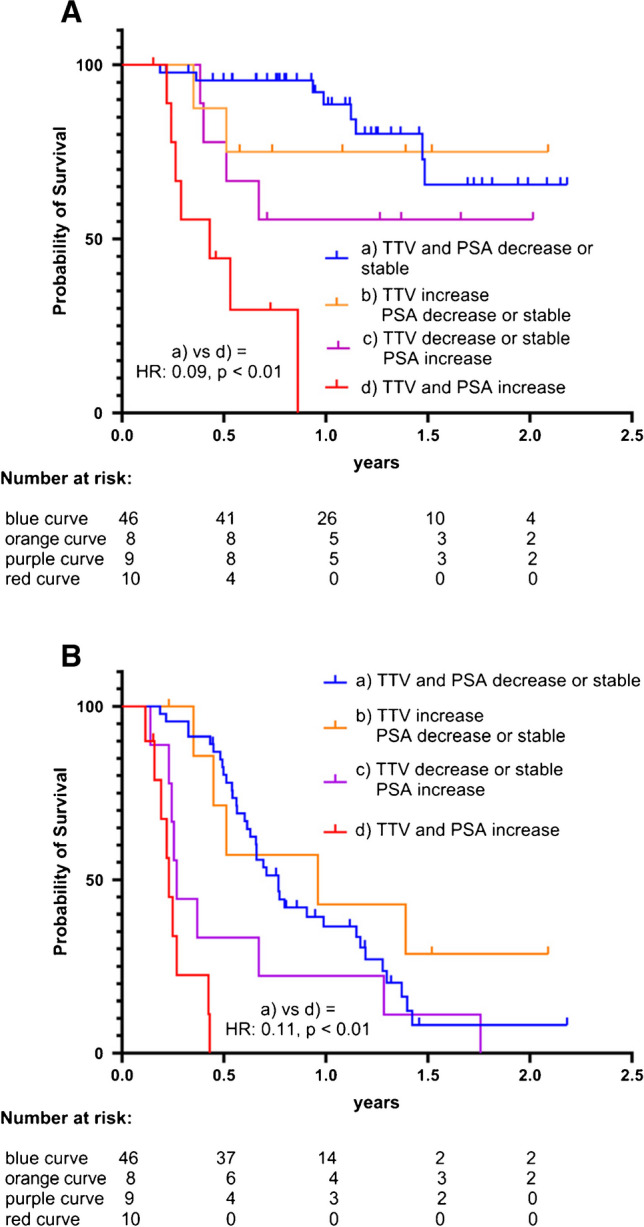
Kaplan–Meier curves for **A** OS and **B** PSA-PFS compared between patients divided into four groups: a) TTV and PSA decrease or stable (blue, *n* = 46), b) TTV increase and PSA decrease or stable (orange, *n* = 8), c) TTV decrease or stable and PSA increase (purple, *n* = 9), d) TTV and PSA increase (red, *n* = 10). Group a) compared to d) with given HR and *p*-values using the Log-rank (Mantel-Cox) test

In a subgroup analysis (Fig. 5), we combined the trends of the PSA values and TTV to establish the following four groups: (a) TTV and PSA decrease or stable (blue, *n* = 46), (b) TTV increase and PSA decrease or stable (orange, *n* = 8), (c) TTV decrease or stable and PSA increase (purple, *n* = 9), (d) TTV and PSA increase (red, *n* = 10).

There was a clear significant increase in OS (*p* < 0.0001, HR 0.09; 95% CI 0.01–0.63) and PSA-PFS (*p* < 0.0001, HR 0.11; 95% CI 0.02–0.68) in the group (a) with a reduced or stable TTV *and* PSA compared to group (d) with a rise in TTV *and* PSA. For the progression group, the median PSA-PFS was 2.8 months and the median OS 5.2 months, whereas in the response group, the median PSA-PFS was 9.2 months and the median OS could not be reached.

### Multivariate analysis

In a multivariate Cox regression analysis, only the TTV change ≤ 0 mL (HR 0.32, 95% CI 0.13–0.76, *p* = 0.01), PSA decrease (HR 0.32, 95% CI 0.13–0.78), and LDH value at baseline (HR for LDH > upper limit or normal 4.5, 95% CI 1.77–12.08, *p* = 0.002) were predictive for OS (supplemental Table 1).

### Correlation between TTV + PSA and only PSA

**Fig. 6:**
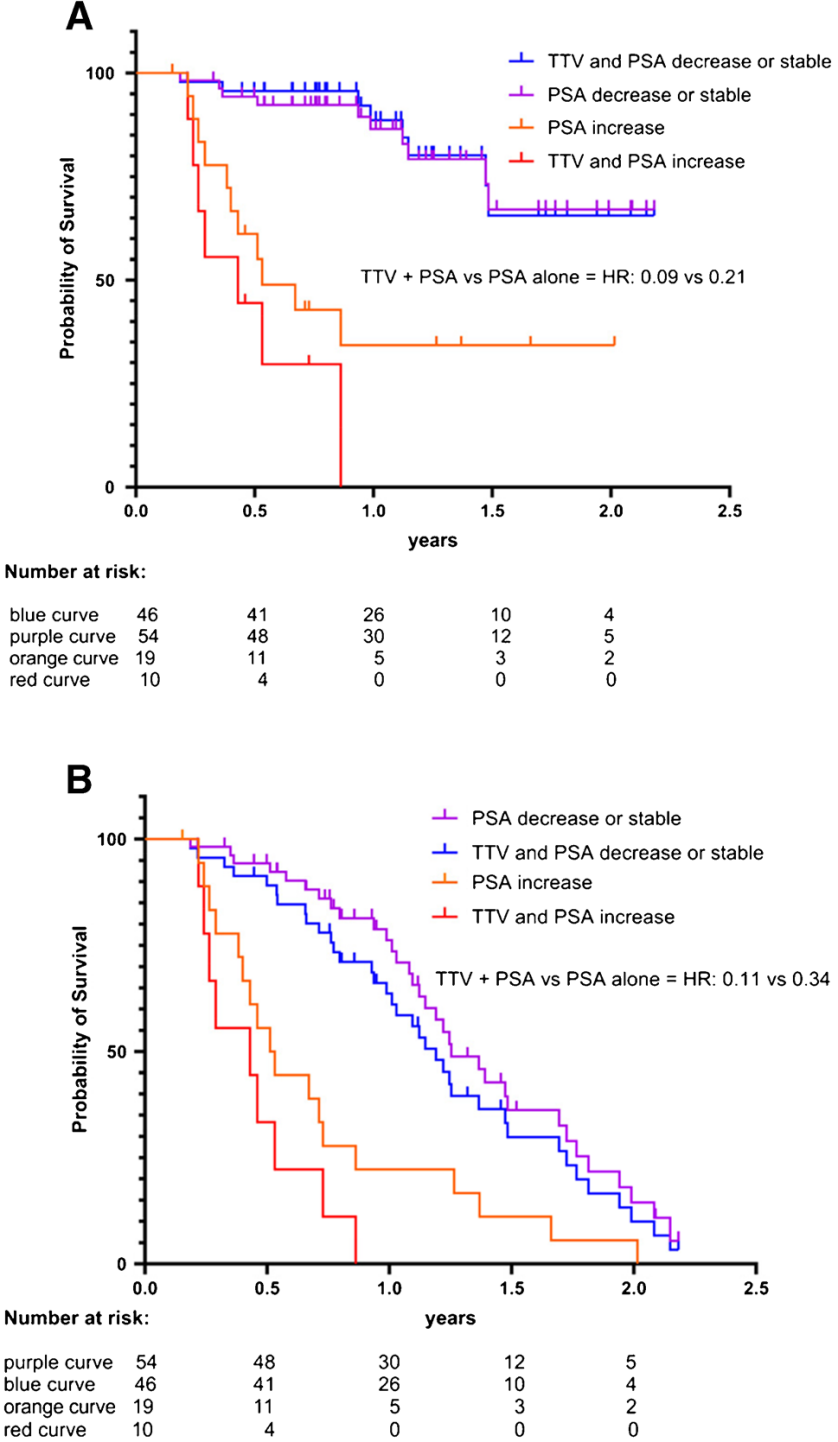
Kaplan–Meier curves for **A** OS and **B** PSA-PFS compared between combined TTV and PSA from Fig. 5 versus PSA alone from Fig. 4. Survival curves of TTV and PSA increase (red) compared to PSA increase alone (orange) as well as TTV and PSA decrease or stable (blue) compared to PSA decrease or stable alone (purple) were not significant different for OS (*p*-values red vs. orange = 0.24; blue vs. purple = 0.88) and PSA-PFS (*p*-values red vs. orange = 0.16; blue vs. purple = 0.31) using the log-rank (Mantel-Cox) test

In a further subgroup (Fig. 6), we compared the results from Fig. 5 of the group (a) (TTV and PSA decrease or stable) and (d) (TTV and PSA increase) to the results from Fig. 4 with PSA alone. Patients with an increase in TTV and PSA had a lower median OS of 5.2 months compared to patients with an increase in PSA alone with an OS of 6.4 months. Yet statistically survival curves of TTV and PSA increase (red) compared to PSA increase alone (orange) as well as TTV and PSA decrease or stable (blue) compared to PSA decrease or stable alone (purple) were not significantly different for OS (*p*-values red vs. orange = 0.24; blue vs. purple = 0.88) and PSA-PFS (*p*-values red vs. orange = 0.16; blue vs. purple = 0.31).

### Correlation between Δ TTV and Δ PSA

The changes in TTV and PSA between the baseline and 6 weeks after showed a reliable positive correlation in our patient cohort with a Pearson index (*r*) of 0.53 (95% CI 0.34–0.68¸ two-tailed *p*-value < 0.0001). In general, absolute TTV values had a positive correlation with PSA levels (*r* 0.36; 95% CI 0.22–0.50, two-tailed *p*-value < 0.0001).

## Discussion

This study shows that change in total tumor burden at cycle 2 (i.e., PSMA-positive tumor volume or TTV, semi-automatically assessed by quantitated SPECT/CT acquired 48 h post-injection of the radiopharmaceutical at baseline and 6 weeks after treatment start) is able to predict overall survival in mCRPC patients receiving [^177^Lu]Lu-PSMA I&T therapy. Therefore, early change in TTV might serve as a biomarker during PSMA-targeted radionuclide therapy and help single out non-responders from responders, as well as stratifying future treatment schemes and thus opening the door to a more personalized treatment approach. While non-responders may benefit from treatment intensification or change, responders may take advantage of a certain treatment modulation (e.g., longer treatment intervals or fewer treatment cycles).

Currently, response criteria include PSA-based endpoints like PSA-progression-free survival defined by PCWG3 and the PSA50 value (PSA decline ≥ 50%) as well as imaging criteria based on RECIST with diagnostic CT and bone scan, which are only evaluated after treatment completion or at the earliest at a predefined interim re-staging. On the contrary, SPECT/CT-based monitoring has the advantage of being done along the treatment course [7]. Additionally, it is known that these established criteria have their limitations [8]. Concerning PSA, there is a large variability of total PSA due to analytical and biological variation, and therefore, a biochemical progression requires significant changes between two consecutive measurements as defined by PCWG3 [9]. Also, though rather rare, PSA-negative metastatic prostate cancers, for which PSA levels are much lower than the tumor burden would suggest, represent a clinical management challenge [10]. Bone metastasis is considered a non-measurable lesion in RECIST, and a sclerotic lesion is often difficult to differentiate between viable tumor, inactive tumor, or benign post-therapeutic changes. Also, bone scintigraphy can be falsely negative and underestimates the volume of disease in case of bone marrow disease or in case no osteoblastic reaction is visible, especially at the beginning, or on the contrary might produce false positive tumor lesions during osteoblastic healing [11].

PSMA-PET/CT has emerged as an accurate imaging modality in prostate cancer; overcoming many of these limitations and its utility is well established in different tumor stages.

Recent work has proposed nomograms using tumor uptake on baseline PSMA-PET/CT, general disease characteristics, and biochemical parameters to prognosticate the outcome after ^177^Lu-PSMA therapy [12]. The change in tumor volumetry from baseline PSMA PET/CT to repeated scan performed after two cycles of therapy (12 weeks) has also been shown to be prognostic for OS [6].

It would be however preferable to be able to make therapy-related predictions and decision based on the easily available post-therapy ^177^Lu-SPECT/CT imaging data in order to avoid additional PET imaging studies and costs. In this sense, Pathmanandavel et al. used quantitated SPECT/CT to assess the changes in total tumor volume (TTV) between baseline and week 12 for mCRPC patients in the LuPIN trial [5]. They reported that, for a subgroup of patients treated with a combination of [^177^Lu]Lu-PSMA-617 and NOX66 2, any increase in TTV was associated with a shorter PSA-PFS. Similar results have been demonstrated by John et al. in a single center retrospective patient cohort undergoing quantitated SPECT/CT at 24 h after [^177^Lu]Lu-PSMA I&T therapy at baseline and at 6 weeks after commencing therapy [4].

In the field of radionuclide therapy, a specific time point for the acquisition of post-treatment SPECT/CT imaging has never been standardized; thus, multiple centers perform these scans at either 24 h or 48 h after therapeutic injections.

Our data evaluates the tumor volumetric changes from baseline to after the first therapy cycle based on quantitated SPECT/CT imaging performed at 48 h after [^177^Lu]Lu-PSMA I&T injection; therefore, we here present relevant information, which is complementary to the current knowledge from previously reported studies. In our cohort, a stable or reduced TTV correlated with a significantly longer OS compared to the group with an increase in TTV. A similar trend was observed concerning the PSA-PFS yet did not reach statistical significance (Fig. 3). Patients without a PSA rise between cycles 1 and 2 (defined as an increase > 2 ng/mL) demonstrated a significant increase in OS and PSA-PFS compared to patients with a PSA rise (Fig. 4).

In a subgroup analysis with combination of the PSA and TTV change, patients with stable or decreasing PSA and TTV had an even more significant difference in OS and PSA-PFS compared to patients with increased PSA and TTV, with a lower hazard ratio than PSA or TTV alone (Fig. 5). In contrast to the published data by John et al. [4], we observed this effect for both OS and PSA-PFS in our patient group instead of only PSA-PFS. In addition, our multivariant analysis showed that TTV and PSA remained statistically significant for OS (supplemental Tab. 1). Survival curves of TTV and PSA combined compared to PSA alone were not significantly different for OS and PFS (Fig. 6); this is to be expected due to the high positive correlation between changes of PSA and TTV. While TTV is not superior to PSA in a direct comparison, the assessment of TTV adds value for treatment monitoring: On the one hand, due to a better split for OS and TTV when combined with PSA versus PSA alone. On the other hand, the semiautomatic assessment of TTV is an easy and fast process, providing additional information from already acquired data. Moreover, the combination of PSA and TTV would also further enhance their reliability by reducing the limitations of each other (e.g., errors of PSA values due to normal variability and errors of TTV values due to technical reasons), since it is unlikely that both parameters produce false positive or false negative results at the same time then one parameter alone. Additionally, it gives relevant information regarding the overall tumor behavior. Especially in patients that show a mixed response with lesions partly increasing and partly decreasing, it provides a surrogate parameter for the overall response of the tumor burden.

Thus, a rise in PSA and TTV after 6 weeks of therapy start might therefore single out patients with only a low chance of treatment response, outweighed by the risk of adverse events.

These findings are also comparable to Gafita et al. who reported a superior prognostic accuracy for OS when combining PSA and PSMA-PET tumor volumetry 12 weeks after the start of therapy compared to PSA alone, making them a possible composite efficacy endpoint for clinical trials of mCRPC [6]. Notably, we could demonstrate this prognostic value at an even earlier therapy stage (6 weeks) with quantitative SPECT/CT without the need for an additional PET/CT scan and previous work showed high and comparable repeatability of tumor volumetry on ^177^Lu-SPECT to PSMA-PET/CT at a minimum SUV cutoff of 3 [5]. This is to emphasize that a treatment adjustment at an earlier time point at 6 weeks after the start of therapy might benefit the patient more than a delayed treatment change after 12 weeks.

We could not observe a significant difference in clinical outcomes between patients with stable or decreased versus increased highest tumor SUVmax or SUVmean. SUV measurements are an indirect marker of PSMA expression density and heterogeneity, which have prognostic value before treatment to assess eligibility and potential chance of treatment benefit but are less suitable for assessing tumor response under therapy since the ligand density does not necessarily correlate with the tumor burden. Therefore, we agree upon the previously stated opinion by Pathmanandavel et al. that PSMA-targeted therapy must focus on changes in volume rather than measures of intensity [5].

The limitations of our study include its retrospective single-center character and a relatively reduced number of patients, possibly responsible for only meeting statistical relevance as an overall prognostic yet not as a therapy response predictor. Furthermore, it is important to emphasize that we assessed the PSMA-positive tumor lesions and therefore might underestimate the whole tumor burden given the possible dedifferentiation of tumor cells in advanced tumor stages. Therefore, diagnostic CT will continue to remain vital in identifying PSMA-negative progression, although by adding intravenous contrast to the CT protocol, the PSMA-positive and PSMA-negative progression might be addressed altogether in one SPECT/CT scan.

In addition, the minimum SUV cutoff value of 3 that was used for the volumetry can be criticized since it is more or less an arbitrary value yet was used by previous ^177^Lu-SPECT volumetry studies [4, 13] and therefore adopted for comparability reasons.

Likewise, we used the same cutoff of any increase instead of a threshold, e.g., reduction of > 30%, since in the previous study by John et al., evidence showed that a threshold of an increase in SPECT-TTV greater than 20% is also associated with PSA-PFS but less significantly than any change in SPECT-TTV [4].

Moreover, we only analyzed the first two SPECT/CTs and evaluation of more time points could provide further insights into the benefit of quantitative ^177^Lu-SPECT in general and TTV in particular. Lastly, while our data support a reevaluation of the treatment regime in patients with increased TTV and PSA after the first cycle, the specifics of treatment change/modification and its predictive value remain uncertain and need a randomized trial powered for outcome.

## Conclusion

Stable or decreased TTV on quantitated SPECT/CT 48 h p.i. appears to be a reliable early prognostic marker after completing the first cycle of [^177^Lu]Lu-PSMA I&T therapy. Combining the TTV changes with PSA levels further increases its prognostic value for OS and renders it significant for therapy response prediction. Additional larger studies using TTV and TTV/PSA are warranted to establish its role as a potential imaging biomarker for personalized treatment approaches.

### Supplementary Information

Below is the link to the electronic supplementary material.

Supplementary file1 (DOCX 18 kb)

## Data Availability

The datasets analysed during the current study are available from the corresponding author in an anonymized form on reasonable request.
