# Supplementary material for: Early response monitoring during [177Lu]Lu-PSMA I&T therapy with quantitated SPECT/CT predicts overall survival of mCRPC patients: subgroup analysis of a Swiss-wide prospective registry study
Source: Eur J Nucl Med Mol Imaging. 2023 Dec 1;51(4):1185–93. doi: 10.1007/s00259-023-06536-2 (PMC10881597; doi:10.1007/s00259-023-06536-2)
Supplement: Supplementary file 1 — Supplementary file1 (DOCX 18 kb) [file 259_2023_6536_MOESM1_ESM.docx]

**Supplemental material**

Multivariate analysis (cox regression)

|  | **HR** | **95 % CI** | **p-value** |
| --- | --- | --- | --- |
| **Age (y)** | 0.99 | 0.94 – 1.04 | ns |
| **Gleason Score** | 1.51 | 0.99 – 2.34 | ns |
| **PSA decrease** | 0.32 | 0.13 – 0.78 | * |
| **Alk. Phos.** | 0.74 | 0.27 – 1.97 | ns |
| **LDH** | 4.54 | 1.77 – 12.08 | ** |
| **TTV decrease** | 0.32 | 0.13 – 0.76 | ** |

**Supplemental Tab. 1:** Multivariate analysis using a cox regression model for OS with the given patient characteristics.
